# Effects of Metformin on Cancer Survival Among Men Diagnosed with Advanced Prostate Cancer Treated with Androgen-Deprivation Therapy: Emulating a Target Trial

**DOI:** 10.3390/cancers17213579

**Published:** 2025-11-06

**Authors:** David S. Lopez, Efstathia Polychronopoulou, Omer Abdelgadir, Raymond Greenberg, Lindsay G. Cowell, Sarah E. Messiah, Yong-Fang Kuo

**Affiliations:** 1 Peter O’Donnell School of Public Health, University of Texas Southwestern Medical Center, Dallas, TX 75390, USA; david.lopez3@utsouthwestern.edu (D.S.L.); raymond.greenberg@utsouthwestern.edu (R.G.); lindsay.cowell@utsouthwestern.edu (L.G.C.); sarah.messiah@utsouthwestern.edu (S.E.M.); 2 Simmons Comprehensive Cancer Center, University of Texas Southwestern Medical Center, Dallas, TX 75235, USA; 3 School of Public and Population Health, University of Texas Medical Branch, Galveston, TX 77555, USA; efpolych@utmb.edu (E.P.); yokuo@utmb.edu (Y.-F.K.)

**Keywords:** metformin, androgen deprivation therapy, advanced stage prostate cancer, survival outcome, target trial emulation

## Abstract

**Background/Objectives:** Metformin is one of the most frequently used concomitant medications among prostate cancer (PCa) patients. However, the effects of metformin on all-cause and PCa-specific mortality among men diagnosed with advanced/metastatic PCa treated with androgen-deprivation therapy (ADT) remain poorly understood, but they may be specifically explained by emulating a target trial. **Methods:** We emulated a target trial of metformin therapy and survival using observational data on 7361 patients diagnosed with advanced PCa, who were treated with ADT, from the Surveillance, Epidemiology, and End Results (SEER)-Medicare database (2008–2019), with completed follow-up until 2020. We included patients with diabetes, and participants were assigned as either “initiator of metformin within 6 months after advanced PCa diagnosis” or “non-initiator of metformin.” We estimated mortality risks using Cox proportional hazards models with adjustment for risk factors via inverse probability weighting using both intention-to-treat and per-protocol analyses. **Results:** Over 13 years of follow-up, with a maximum 3 years of follow-up after PCa diagnosis, all-cause mortality occurred in 52 metformin initiators (47.7%) versus 3052 non-initiators (42.1%), while PCa-specific mortality occurred in 36 initiators (33.0%) versus 1919 non-initiators (26.5%). In the intention-to-treat analysis, metformin initiation was not associated with all-cause mortality (Hazard Ratio [HR] = 1.38, 95% CI: 0.98–1.95) or PCa-specific mortality (HR = 0.99, 95% CI: 0.63–1.55). Similarly, in per-protocol analysis, there was no evidence of risk reduction with all-cause (HR = 1.20, 95% CI = 0.80–1.81) or PCa-specific mortality (HR = 1.45, 95% CI = 0.88–2.38) after adjusting for time-varying covariates and allowing a 30-day gap for metformin discontinuation, adjusted for via inverse probability weighting. **Conclusions:** Our findings align with prior randomized trials showing no survival benefit of metformin in advanced PCa patients receiving ADT. Timing of metformin discontinuation also showed no significant effect. However, the small size of the metformin initiator group precluded subgroup analyses for hormone-sensitive (HSPC) and castrate-resistant prostate cancer (CRPC), limiting our ability to explore potential differential effects.

## Simple Summary

Prostate cancer has a 99% five-year survival rate, but this drops to 34% in advanced cases, highlighting the need for better treatment strategies. This study evaluated the impact of metformin use on all-cause and PCa-specific mortality among men with advanced PCa undergoing androgen-deprivation therapy (ADT). Using data from the SEER-Medicare database (2008–2019), we emulated a target trial involving 7361 diabetic patients, comparing those who initiated metformin within 6 months of diagnosis to non-initiators. Mortality outcomes were assessed using pooled logistic regression, adjusted for confounders through inverse probability weighting (IPW), under both intention-to-treat and per-protocol frameworks. The results indicated no significant association between metformin use and reduced all-cause or PCa-specific mortality. These findings remained consistent even after accounting for treatment adherence and timing of discontinuation. Overall, the study aligns with prior randomized trials, suggesting metformin does not confer a survival benefit in the context of advanced PCa managed with ADT.

## 1. Introduction

The five-year survival rate for prostate cancer (PCa) is extremely high (99%). However, advanced PCa has a markedly different prognosis with only a 34% five-year survival rate [1], highlighting the need for focused clinical attention on advanced cases. PCa is the second most common cause of cancer death among men in the United Stated (US), accounting for 11% of all cancer deaths, with a mortality rate that rises steeply with age and shows no peak [2]. At ages 50–54 and 55–59, mortality rates are 3.1 and 8.5/100,000 men per year, respectively, whereas at ages 75–79, 80–84, and ≥85 years, the rates are 126.3, 219.9 and 461.8/100,000, respectively [3]. Thus, PCa is an important cancer for men as they age, with the greatest burden of death among older men [3]. New PCa therapies have been developed and approved by the Federal Drug Administration (FDA) [4], yet nearly 34,500 men died of PCa in 2022 [2]. Men diagnosed with advanced PCa are traditionally treated with androgen-deprivation therapy (ADT) [5]. The primary benefit of ADT is disease regression; however, it does not provide a cure, as hormone-insensitive disease often develops from resistant clones [6].

Metformin is commonly used as the first line of medication among older men (≥65 yrs old) with type 2 diabetes [7]. Furthermore, metformin has also been investigated for the treatment of various cancers, and it remains as one of the most frequently used concomitant medications among PCa patients [8,9,10]. The use of metformin as an adjuvant therapy is based largely on biological evidence from experimental studies demonstrating anticancer properties [11,12,13]. Yet the findings from observational studies [8,14,15,16,17,18] and randomized controlled clinical trials (RCT) [9,19,20,21] investigating the effects of metformin on cancer remain inconsistent, which is due, in part, to the investigation of different cancer outcomes (e.g., total cancer, stage [localized vs. advanced stage], grade [low vs. high], and survival), and the selection of study designs (e.g., prospective/retrospective cohort and RCT) [8]. Thus, there remains a paucity of research focused on the effects of metformin on all-cause and PCa-specific mortality among men diagnosed with advanced PCa treated with ADT.

PCa is a heterogenous disease, where risk factors for indolent PCa differ from those for advanced and fatal PCa [22]. Studies investigating the association of metformin with incident total PCa, which combines stage (localized vs. advanced [III and IV]) and grade (low vs. high [≥8]), have also yielded mixed results [8,15,16,17,18,21]. A recent emulated target trial that leveraged electronic health records and focused on the association of metformin with incident total PCa reported null results [23]. However, this latter study also combined PCa stage and grade. Of interest is the emerging body of literature suggesting that metformin has potential benefits to cancer survival mainly among men diagnosed with advanced PCa (stage III and IV) who have received standard PCa therapy such as ADT [9,14,19]. Among men with advanced PCa receiving ADT, treatment-induced metabolic disturbances, particularly insulin resistance and hyperinsulinemia, may contribute to disease progression and adversely affect survival. Metformin, through its activation of AMP-activated protein kinase (AMPK) [24], inhibition of the mechanistic target of rapamycin (mTOR) pathway [25], and reduction in insulin/insulin-like growth factor 1 (IGF-1) signaling [26], offers a biologically plausible mechanism for mitigating these effects. Given these considerations and the elevated mortality risk in this population, we emulated a target trial to investigate the association between metformin use and both all-cause and PCa–specific mortality among men diagnosed with advanced/metastatic PCa treated with ADT, while accounting for selection- and time-related biases inherent in observational data.

## 2. Materials and Methods

### 2.1. Specification of the Target Trial

#### 2.1.1. Study Design

We designed this study by leveraging observational data to emulate a hypothetical randomized trial assessing the effectiveness of metformin on survival after diagnosis with advanced PCa. The design adhered to the recently published Transparent Reporting of Observational Studies Emulating a Target Trial (TARGET) guidelines [27]. The key components of the target trial are summarized in Table 1.

#### 2.1.2. Participants

Eligibility criteria for the target trial included age ≥ 66 years at diagnosis with advanced or metastatic PCa between 2008 and 2019 and receipt of ADT within 6 months from diagnosis. Additional criteria were no metformin use in the 6 months prior to diagnosis (with continuous Part D enrollment), no metformin contraindication (hepatic failure, severe renal disease, metabolic acidosis, and chronic heart failure), at least one year of prior health information available (Parts A and B without Health Maintenance Organizations [HMO]) and alive and enrolled in Part D for at least 6 months post-diagnosis.

#### 2.1.3. Procedures

The treatment strategies compared were (a) initiation of metformin therapy within 6 months of diagnosis and continuous use over follow-up until the development of a contraindication or death and (b) no initiation of metformin until the development of an indication (type 2 diabetes). The outcomes of interest are all-cause mortality and PCa-specific mortality within 3 years from diagnosis. Of note, cancer-specific mortality data in SEER-Medicare are derived from the National Center for Health Statistics (NCHS) and linked to cancer incidence records [28]. The SEER cause-specific death classification system uses an algorithm that assigns a single underlying cause of death based on tumor characteristics and comorbidities. While this approach is widely accepted and validated in prior studies, it remains imperfect. Misclassification is inevitable given the known limitations of death certificate data. For example, deaths are sometimes attributed to metastatic sites rather than the primary cancer. This potential for misclassification warrants caution in interpreting cause-specific mortality findings.

In the analysis plan, baseline (time zero) for each eligible individual was defined as the date when all eligibility criteria were satisfied, which also marked the initiation of follow-up. This corresponded to the date of both ADT and metformin initiation for treatment strategy (a), or the date of ADT initiation for treatment strategy (b). Follow-up ends at the end of the observation period (3 years after diagnosis), administrative end of study (31 December 2020), loss to follow-up, or death, whichever occurs first. Intention-to-treat and per-protocol analyses for adherence to treatment strategy are used to estimate the effect of interest. In the per-protocol analysis individuals are censored when they deviate from their assigned treatment strategy. Time-varying inverse probability weights are used to estimate the monthly probability of adherence to treatment adjusting for baseline and time-varying covariates.

### 2.2. Emulation of the Target Trial

#### 2.2.1. Data Source

We emulated the target trial described using 2008–2019 data from Surveillance, Epidemiology, and End Results (SEER)-Medicare, a linkage of population-based cancer registries from 19 SEER regions with longitudinal Medicare administrative data [29]. The SEER program collects detailed demographic characteristics, incidence, and survival information from population-based cancer registries [30]. The Medicare database contains comprehensive information on healthcare services for individuals 65 years and older. Medicare claims are linked through unique SEER identifiers, covering the time of Medicare eligibility until death. We used this observational dataset to emulate each target trial feature as closely as possible, as mentioned in Table 1. The Institutional Review Board (IRB) of the University of Texas Medical Branch (Galveston, TX, USA) approved this study (Approval Code: 20-0237; date: 15 September 2020).

#### 2.2.2. Variables, Endpoints, and Follow-Up

**Figure 1:**
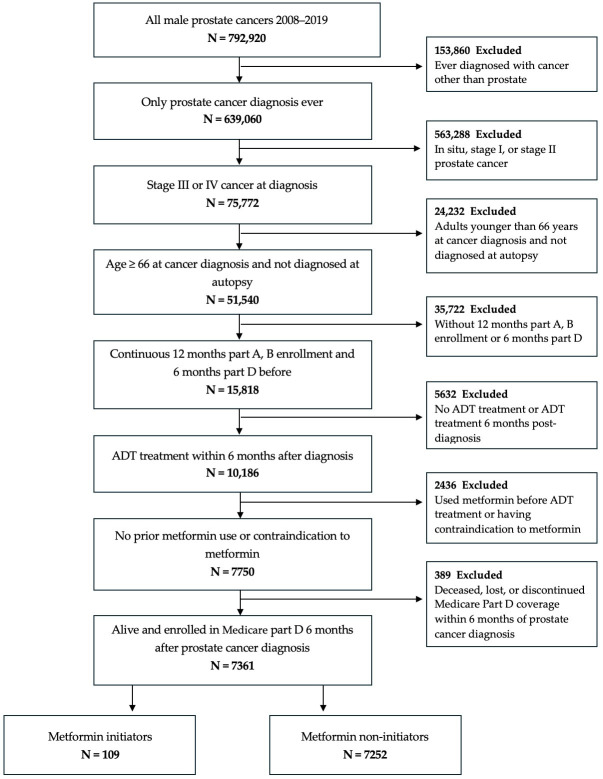
Flowchart of eligible individuals, SEER-Medicare 2008–2019.

Demographics, clinical information, and all-cause mortality and PCa-specific mortality were collected from the SEER cancer registry. Patients were followed at baseline date when all eligibility criteria are met until whichever occurred first: observation period (3 years after diagnosis), administrative end of study (31 December 2020), loss to follow-up, or death. Poverty and education levels were determined at the zip-code level and categorized in quartiles. Prescriptions of metformin were identified from National Drug Codes (NDC) using the 2024 Redbook Select database (RED BOOK Select Extracts, Truven Health Analytics). A gap of up to 30 days between the end of a prescription’s supply and the next filled prescription was allowed when determining continuous metformin use. ADT was defined as receiving hormonal prescriptions or having an orchiectomy within 6 months from diagnosis and was determined using NDC and Current Procedural Terminology (CPT) codes. Indications and contraindications to metformin as well as comorbidities were determined using International Classification for Diseases (ICD) versions 9 and 10 codes. All comorbidities, metformin indication, and contraindications were assessed in the year prior to cancer diagnosis as well as at each month of follow-up. Figure 1 shows a flowchart of the selection and flow of eligible patients.

#### 2.2.3. Statistical Analysis

Demographic and clinical characteristics by metformin initiation status are described as frequency and percentages or means and standard deviation for categorical and continuous variables, respectively, and compared with chi-square or *t*-test (or non-parametric equivalent) as appropriate. We estimated the observational analog of the intention-to-treat effect by using a Cox proportional hazards model, adjusting for baseline covariates to emulate randomization, and by incorporating stabilized inverse probability weights (IPW) to adjust for pre-diagnostic prognostic factors associated with probability of treatment (Table 1). For the per-protocol observational analog effect, we used a weighted, pooled logistic model for the monthly probability of death, adjusted for covariates and incorporating stabilized inverse probability weights for pre- and post-diagnostic prognostic factors associated with adherence to treatment assignment. IPW were truncated at their 99th percentile to minimize the influence of outliers, and robust standard errors were calculated for the model parameters.

#### 2.2.4. Sensitivity Analysis

To minimize misclassification and selection bias, we broadened the prescription gap window from 30 to 60 days when defining continuous metformin use. This change reflects real-world clinical practice, where brief treatment interruptions often occur without stopping therapy. By allowing a longer gap, we captured these interruptions more accurately, resulting in a slightly larger and more representative sample. Clinically, this approach prevents overestimating non-adherence and better aligns with how patients actually use medications. We also incorporated additional weights to account for loss to follow-up in the per-protocol analysis. All analyses were conducted using SAS software (v.9.4, SAS Institute, Cary, NC, USA).

## 3. Results

Table 2 shows the baseline characteristics of the 7361 individuals eligible for emulating the target trial among individuals diagnosed with advanced stage PCa, who were treated with ADT. When we compared with metformin non-initiators at baseline, metformin initiators were, in general, more likely to be Hispanic or Non-Hispanic (NH) Black men, and had a higher frequency of ≥3 Charlson Comorbidity index and higher prevalence of diabetes.

In the emulated target trial, Table 3 shows the risk of dying (all-cause and prostate cancer-specific) among metformin initiators and non-initiators in the intent-to-treat and per-protocol analyses after adjusting for age, race/ethnicity, marital status, percent of adults with less than high school education, Charlson Comorbidity index, diabetes, congestive heart failure (CHF), cerebrovascular disease, hypertension (HTN), depression, and number of primary care physician (PCP) visits in the year before diagnosis; in the per-protocol analysis we additionally adjusted for the month of follow-up and its square term. In the intent-to-treat analysis, the hazard ratio for all-cause mortality was 1.389 (95% CI = 0.987–1.955) and for PCa-specific mortality was 0.997 (95% CI = 0.636–1.556). Similarly, in the per-protocol analysis, the hazard ratio for all-cause mortality was 1.068 (95% CI = 0.688–1.660) and for PCa-specific mortality was 1.417 (95% CI = 0.868–2.321), after censoring individuals when they (n = 102) deviated from their assigned treatment strategy (30-day gap).

Sensitivity analyses were conducted for potential misclassification and selection bias due to censoring for loss to follow-up and treatment discontinuation in Table 4. We first estimated the observational analog of the per-protocol effect after applying additional weights for censoring due to loss to follow-up (n = 117) showed a hazard ratio of 1.038 (95% CI = 0.663–1.625) for all-cause mortality, and a hazard ratio of 1.379 (95% CI = 0.835–2.276) for PCa-specific mortality. In further analyses, we allowed a 60-day gap, instead of a 30-day gap, for metformin discontinuation (n = 17), and it showed a hazard ratio of 1.229 (95% CI = 0.852–1.773) for all-cause mortality, and a hazard ratio of 1.401 (95% CI = 0.923–2.125) for PCa-specific mortality (Table 4).

## 4. Discussion

After emulating a target trial of 7361 individuals leveraging the SEER-Medicare claims data, our findings suggest that metformin therapy initiation does not improve the survival of patients. After addressing potential selection, confounding, and immortal time biases, metformin therapy did not influence the risk of all-cause and PCa-specific mortality among advanced stage PCa patients treated with ADT. These findings are consistent with the results of previous pooled analyses of randomized trials investigating survival among PCa patients treated with ADT, but different to the ones from observational studies using a similar advanced PCa population, mortality, and metformin initiator.

Our null effects with all-cause mortality are similar to two previous RCTs that investigated the effects of metformin on overall survival among advanced PCa participants treated with ADT [9,19], yet inconsistencies remained [9]. In the pooled analysis of three RCTs with 889 metformin initiators, the treatment of metformin was not associated with overall survival among advanced PCa patients (HR = 0.83, 95% CI’s = 0.67–1.03) [9]. Similar null effects were observed in a single blinded RCT with 62 metformin initiators (MANSMED) [19]. However, one of the RCTs in the pooled analysis (PREVAIL, n = 182 metformin initiators), the radiographic prostate cancer-free survival, a surrogate for overall survival [31], was improved after comparing metformin initiators with non-initiators (HR = 0.48, 95% CI’s = 0.34–0.70). These differences between our emulated target trial, previous pooled analyses of RCTs, and the single PREVAIL RCT can be attributed, in part, to the different responses to ADT-plus-metformin treatment for hormone-sensitive prostate cancer (HSPC) and castrate-resistant prostate cancer (CRPC). HSPC responds well to treatment, but CRPC is more aggressive, painful, and resistant to therapies [32]. Due to our limited sample size with metformin initiators and advanced PCa cases, we did not explore differences between HSPC and CRPC in relation to ADT treatment. Therefore, the findings of our study should be interpreted under those limitations.

Furthermore, a previous meta-analysis of RCTs (n = 9 studies) that focused on the effects of overall survival in relation to metformin treatment plus anticancer therapy (e.g., gemcitabine/erlotinib/paclitaxel/carboplatin) among advanced or metastatic cancer (total cancer) patients reported null effects (HR = 0.97, 95% CI’s = 0.80–1.16) [20]. Although there is a biological and clinical difference between overall survival among advanced PCa patients treated with ADT and the incidence of total PCa, it is interesting to note that a meta-analysis of two RCTs also reported null results on the incidence of total PCa in relation to metformin treatment (OR = 1.18, 95% CI’s = 0.69–2.04) [21].

Most RCTs are reporting null effects on overall and cancer-specific survival in relation to metformin treatment among advanced PCa patients treated with ADT [9,19,21]. Because our emulated target trial focused on defining time zero by properly aligning eligibility criteria, treatment assignment, and start of follow-up time, our findings agree with the previous RCTs [33]. However, observational studies report different results. A large study that leveraged a national Veteran Affairs database found improved overall and PCa-specific survival among advanced PCa men treated with ADT using metformin (overall survival HR = 0.82, 95% CI’s = 0.78–0.86; PCa-specific survival HR = 0.70, 95% CI’s = 0.64–0.77, respectively) [14]. Another small study of metformin users (n = 132) showed that metformin remained a significant predictor of favorable overall survival (HR = 0.55, 95% CI’s = 0.32–0.96) after adjusting for high-grade and advanced-stage of PCa. Similarly, a meta-analysis of observational studies conducted among early-stage PCa patients reported that metformin has benefits in overall survival (HR = 0.82, 95% CI’s = 0.73–0.93; n = 4 studies) and PCa-specific survival (HR = 0.58, 95% CI’s = 0.37–0.93; n = 3 studies) [15]. Our emulated target trial and the previous pooled analyses of RCTs [9] are similar to the large national Veterans Affair study (observational study) [14] in terms of research objectives, exposures, outcomes and PCa population (effects of metformin initiation on overall and cancer-specific survival among advanced PCa patients treated with ADT), but our findings, as well as the pooled analyses of RCTs, were null. It is possible that this difference could be, in part, because the Veterans Affairs study could not account for reasons of drug discontinuation, and the definition of patients for whom ADT was initiated for HSPC and CRPC.

A combination of methodological (alignment of eligibility criteria, treatment assignment and start of follow-up) and biological/clinical factors (response of HSPC and CRPC to ADT and metformin treatments) could be the driving force in the different findings when RCTs, emulated target trial frameworks, and observational studies (prospective and retrospective cohort studies) are compared with each other. This includes considering the discontinuation of metformin treatment in our target trial at a 30-day gap and 60-day gap.

Our study had some limitations. First, our study was not able to differentiate between HSPC and CRPC due to a limited sample size [32,34]. This differentiation is important because treatment for advanced PCa with ADT serves as the foundational treatment, but it is not sufficient for long-term control [34]. Therefore, ADT treatment has been combined with other chemotherapies (e.g., docetaxel) or androgen pathway-directed therapy (e.g., enzalutamide), and it has been reported to have the potential to influence overall survival [6,35]. Although we did not find a benefit to overall survival, future studies should explore these ADT combined therapies. Second, we adjusted for several potential baseline and time-varying confounders for metformin treatment and mortality [36], but we were not able to adjust for dietary intake, physical activity, sleep, and other pertinent laboratory information due to limited data availability. SEER-Medicare indicates that laboratory results or other environmental, and nutritional/lifestyle (e.g., smoking) factors are incompletely reported with low sensitivity. This includes data for body mass index (≥30 kg/m^2^) to define obesity, which could be a risk factor for advanced PCa and mortality. However, in a previous sensitivity analysis investigation we adjusted our multivariable models by including obesity, using diagnostic ICD-10 codes (e.g., E66.01, .2, .3, and Z68/35-39), and the results remained nearly identical to the ones without obesity included due to the low sensitivity of SEER-Medicare data [37]. Furthermore, although we are emulating a target trial, this is an observational study, so the assignment to a treatment strategy was not randomized. Therefore, if unknown confounders are unequally distributed within our comparison groups, there may be residual confounding, and our findings should be interpreted with caution. Third, there are inherent general limitations of leveraging Medicare claim data, such as possible coding errors, omissions of claims [29], or the use of medications prior to 2007 (earliest year of available Part D data). However, any potential inaccuracy in the data will be considered nondifferential misclassification, as it was collected prior to the disease onset, generally biasing associations toward the null (1.0). Fourth, the limited number of metformin initiators reduces the statistical power to detect meaningful differences in mortality. Our post hoc power calculation was 0.617, below the conventional threshold for adequate power. To reliably detect the observed effect size, approximately 170 additional participants would be required in this group. As it stands, the findings should be validated with adequately powered studies before drawing firm conclusions. Fifth, we used zip-code-level poverty and education as proxies for individual socioeconomic status (SES). Though common in observational studies, this approach risks ecological fallacy, assuming neighborhood traits apply uniformly to individuals. Socioeconomic variation within zip codes is substantial, so SES adjustment is likely incomplete, and residual confounding remains possible. Future studies should prioritize individual-level SES data over geographic surrogates, which often reflect convenience more than precision. Finally, our study population included ≥65-year-old patients with Medicare claims, so our results may not be generalizable to research cohorts using other types of insurance, no insurance at all, or a younger population.

## 5. Conclusions

Our emulated target trial findings suggest that there is no potential benefit of adjuvant metformin treatment on death reduction among advanced/metastatic PCa patients treated with ADT. In per-protocol analysis, metformin treatment discontinuation at different times, 30 days or 60 days, yielded similar null results. This observation is consistent with previous studies addressing similar exposure (s), outcome (s), and study population.

## Figures and Tables

**Table 1:** Summary of target trial specification and emulation characteristics.

	Target Trial Specification	Emulating Target Trial
**Causal question**	Does metformin initiation, compared to no initiation, affect the risk of all-cause and prostate cancer (PCa)-specific mortality in patients with an advanced PCa diagnosis who have been treated with androgen-deprivation therapy (ADT)?	Same as for the target trial.
**Eligibility criteria**	• Advanced PCa diagnosis 2008–2019. • Age ≥ 66 at diagnosis. • ADT within 6 months • No prior metformin used in the past 6 months. • No metformin contraindication. • One-year health information pre-diagnosis. • Alive at 6 months after diagnosis.	Same as for the target trial. 1-year prior health information availability was determined by having continuous Medicare parts A and B enrollment in the year before. No prior metformin used required at least 6 months of Medicare part D enrollment prior to diagnosis. All variables were identified using National Drug Codes (NDC), Current Procedural Terminology (CPT) codes., and International Classification for Diseases (ICD) versions 9 and 10 codes.
**Treatment strategies**	(a) Initiation of metformin at baseline and continuation until a contraindication develops or follow-up ends. (b) Non-initiation of metformin at baseline or over follow-up unless an indication develops.	Same as for the target trial. Metformin was identified by NDC.
**Treatment assignment**	Individuals are randomly assigned to a treatment strategy at baseline and are aware of the strategy to which they have been assigned.	Because randomization is not feasible in observational settings, individuals are classified to a strategy based on their metformin prescription data in the 6 months after diagnosis.
**Outcomes**	All-cause and PCa-specific mortality, occurring within 3 years from diagnosis.	Same as for the target trial.
**Follow-up initiation**	Starts on the day of treatment assignment (baseline).	Follow-up begins at the baseline date (time zero), defined as the date in which all eligibility criteria are met (both ADT and metformin have initiated for treatment strategy a, or just ADT for treatment strategy b).
**Follow-up endpoint**	The earliest occurrence of death, loss of follow-up, 3 years after diagnosis, or at the administrative end date of follow-up.	The end of follow-up is defined similarly as the earliest occurrence of death, loss of follow up, 3 years after diagnosis, or at the administrative end date of follow-up, which is 31 December 2020
**Causal contrasts**	Intention-to-treat and per-protocol effects.	The emulation estimates the observational analog of the intention-to-treat and per-protocol effects. The resulting association reflects the hazard ratio for all-cause and PCa-specific death.
**Statistical analysis**	Descriptive statistics. Hazard ratios (HRs) and their 95% confidence intervals (CIs) for the risk of death are estimated using Cox proportional hazards models for intention-to-treat and per-protocol effects.	Descriptive statistics. HRs with 95% CIs for death in the intention-to-treat analysis were estimated using Cox proportional hazards models, applying inverse probability weighting (IPW) to adjust for baseline factors associated with treatment probability. For the per-protocol analysis, HRs with 95% CIs for death were estimated using Cox proportional hazards models, censoring individuals at the time they deviated from their assigned treatment strategy, and applying IPW to adjust for both baseline and time-varying factors related to treatment adherence.

**Table 2:** Characteristics of the 7361 Eligible Individuals, SEER-Medicare 2008–2019.

Characteristics	Category	Non-Initiators n = 7252 (%)	Metformin Initiators n = 109 (%)	*p*-Value
Age, mean (SD)		75.4 (6.8)	74.3 (6.4)	0.0964
Race/ethnicity	Hispanic	512 (7.06)	13 (11.93)	0.0074 *
	Non-Hispanic Black	608 (8.38)	17 (15.6)	
	Non-Hispanic White	5655 (77.98)	>68 (>66.97)	
	Other	477 (6.58)	<11 (<5.5) **	
Marital status	Divorced/Widowed	1149 (15.84)	11 (10.09)	0.4403
	Married	4749 (65.49)	>73 (>69.72)	
	Never Married	843 (11.62)	14 (12.84)	
	Unknown	511 (7.05)	<11 (<7.34) **	
Percent of adults with less than high school education	1st quartile	1933 (26.65)	29 (26.61)	0.0657
	2nd quartile	1748 (24.1)	25 (22.94)	
	3rd quartile	1627 (22.44)	24 (22.02)	
	4th quartile	1359 (18.74)	>21 (>26.61)	
	Unknown	585 (8.07)	<11 (<1.83) **	
Percent of households below poverty	1st quartile	1854 (25.57)	25 (22.94)	0.0744
	2nd quartile	1727 (23.81)	>23 (>29.36)	
	3rd quartile	1603 (22.1)	22 (20.18)	
	4th quartile	1483 (20.45)	28 (25.69)	
	Unknown	585 (8.07)	<11 (<1.83) **	
Rural/urban residence	Metro	>5957 (>82.28)	>78 (>79.82)	0.8628
	Rural	162 (2.23)	<11 (<1.83) **	
	Urban	1122 (15.47)	20 (18.35)	
	Unknown	<11 (<11) **	0 (0)	
Charlson Comorbidity index	0	4433 (61.13)	47 (43.12)	0.0011 *
	1	1487 (20.5)	33 (30.28)	
	2	725 (10)	13 (11.93)	
	3 or more	607 (8.37)	16 (14.68)	
Diabetes	No	6483 (89.4)	59 (54.13)	0.0001 *
	Yes	769 (10.6)	50 (45.87)	
CHF	No	6998 (96.5)	>98 (>95.41)	0.5418
	Yes	254 (3.5)	<11 (<4.59) **	
Cerebrovascular disease	No	6770 (93.35)	>98 (>93.58)	0.9256
	Yes	482 (6.65)	<11(6.42) **	
Hypertension	No	2370 (32.68)	31 (28.44)	0.3486
	Yes	4882 (67.32)	78 (71.56)	
Depression	No	6690 (92.25)	98 (89.91)	0.3650
	Yes	562 (7.75)	11 (10.09)	
Number of PCP visits in the year before diagnosis, mean (SD)		4.9 (5.5)	5.0 (5.7)	0.6166
All-cause mortality at any time	No	4200 (57.92)	57 (52.29)	0.2381
	Yes	3052 (42.08)	52 (47.71)	
PCa-specific mortality at any time	No	5333 (73.54)	73 (66.97)	0.1234
	Yes	1919 (26.46)	36 (33.03)	

Abbreviations: CHF, Congestive heart failure; PCa, Prostate cancer; PCP, Primary care physician; SD, Standard deviation. * Statistical significance at the *p* value < 0.05 level. ** SEER-Medicare data presentation guidelines have been followed. In accordance with the Data Use Agreement, all unweighted counts below 11 have been suppressed to prevent the potential identification of individuals based on unique or rare combinations of characteristics.

**Table 3:** Hazard ratios for all-cause- and prostate cancer-specific mortality comparing metformin initiators vs. non-initiators among advanced-stage PCa patients treated with ADT, SEER-Medicare 2008–2019.

Metformin Initiators vs. Non-Initiators		
**Intent-to-Treat**	**Hazard Ratio (HR)**	**95% Confidence Interval (CI)**
All-cause mortality	1.389	0.987–1.955
PCa-specific mortality	0.997	0.636–1.556
**Per-Protocol ^A^**	**Hazard Ratio (HR)**	**95% Confidence Interval (CI)**
All-cause mortality	1.068	0.688–1.660
PCa-specific mortality	1.417	0.868–2.321

Cox models adjusted for age, race/ethnicity, marital status, percent of adults with less than high school education, Charlson Comorbidity index, diabetes, CHF, cerebrovascular disease, HTN, depression, and number of PCP visits in the year before diagnosis. ^A^ Patients who deviated from metformin treatment (30 day-gap), monthly inverse probability weighting, time-varying comorbidities.

**Table 4:** Per-protocol-sensitivity analysis: hazard ratios for all-cause and prostate cancer-specific mortality comparing metformin initiators vs. non-initiators among advanced-stage PCa patients treated with ADT, SEER-Medicare 2008–2019.

Metformin Initiators vs. Non-Initiators		
**Per-Protocol ^A^**	**Hazard Ratio (HR)**	**95% Confidence Interval (CI)**
All-cause mortality	1.038	0.663–1.625
PCa-specific mortality	1.379	0.835–2.276
**Per-Protocol ^B^**	**Hazard Ratio (HR)**	**95% Confidence Interval (CI)**
All-cause mortality	1.229	0.852–1.773
PCa-specific mortality	1.401	0.923–2.125

Cox models adjusted for age, race/ethnicity, marital status, percent of adults with less than high school education, Charlson Comorbidity index, diabetes, CHF, cerebrovascular disease, HTN, depression, and number of PCP visits in the year before diagnosis. ^A^ Per-protocol effect with weights for loss of follow-up censoring in addition to treatment deviation (30 day-gap). ^B^ Per-protocol effect with 60-day gap for defining metformin discontinuation.

## Data Availability

The SEER-Medicare datasets used to conduct this study are available upon approval of a research protocol by the National Cancer Institute. Instructions for obtaining these data are available at https://healthcaredelivery.cancer.gov/seermedicare/obtain/ (accessed on 9 March 2024).

## Abbreviations

The following abbreviations are used in this manuscript:

ADT	Androgen-deprivation therapy
AMPK	AMP-activated protein kinase
CHF	Congestive heart failure
CI	Confidence Interval
CRPC	Castrate-resistant prostate cancer
CPT	Current Procedural Terminology codes
FDA	Federal Drug Administration
HR	Hazard Ratio
HSPC	Hormone-sensitive prostate cancer
HTN	Hypertension
ICD	International Classification for Diseases
IGF-1	Insulin/insulin-like growth factor 1
IPW	Inverse probability weights
IRB	Institutional Review Board
mTOR	Mechanistic target of rapamycin
NDC	National Drug Codes
NH	Non-Hispanic
PCa	Prostate cancer
PCP	Primary care physician
RCT	Randomized controlled clinical trials
SD	Standard deviation
SEER	Surveillance, Epidemiology, and End Results
SES	Socioeconomic status
